# Effects of Lifestyle Modifications and Dietary Habits on Prevention of Diabetes and Cardiovascular Disease

**DOI:** 10.1155/2018/2341230

**Published:** 2018-12-11

**Authors:** Samy I. McFarlane, Azizi A. Seixas, Charles Agyemang, Girardin Jean-Louis

**Affiliations:** ^1^College of Medicine, Department of Medicine, Division of Endocrinology, State University of New York, Downstate Medical Center, Brooklyn, NY, USA; ^2^Department of Population Health and Psychiatry, New York University School of Medicine, New York, NY, USA; ^3^Department of Public Health, Amsterdam Public Health (APH) Research Institute, Amsterdam University Medical Centres, Meibergdreef 9, 1105 AZ Amsterdam, Netherlands

Diabetes is a major public health problem affecting millions of people around the globe. According to the International Diabetes Federation (IDF) global estimates in 2015, 1 in 11 persons had diabetes (around 415 million people). This represents a significant increase from 285 million reported in 2010 [1]. The report also indicates that the observation of 1 death every 6 seconds may be attributed to diabetes or diabetes-related complications [1]. In the United States, an estimated 29.1 million Americans or 9.3% of the population have diabetes and it is estimated that another 27.8% are undiagnosed [2, 3]. The prevalence of diabetes increases significantly with age, affecting around 16.2% of American adults aged 45 to 64 years and 25.9% of adults aged 65 years or older; prevalence is also affected by increased obesity particularly central obesity [2–5]. The Centers for Disease Control and Prevention (CDC) estimates that, with current trends, as many as 1 in 3 American adults could have diabetes by 2050 [3]. Ethnic minority groups and migrant populations in high-income countries have been particularly affected [6, 7]. A systematic review in Europe shows that odds is 6-fold higher among some ethnic groups compared with the European host populations.

In light of the increasing prevalence of diabetes and cardiometabolic conditions globally, more comprehensive preventive programs are needed to curb them. Unfortunately, to our knowledge, there has not been a systematic and comprehensive collection of scientific evidence on biobehavioral mechanisms that underlie diabetes and cardiometabolic conditions. The current special issue is intended to fill this important gap and presents comprehensive evidence on biological and behavioral factors that are associated or engender diabetes and cardiometabolic conditions.

Risk factors for diabetes include nonmodifiable and potentially modifiable factors (Table 1). Among the most important potentially modifiable risk factors for diabetes are sedentary lifestyle, poor dietary habits, and sleep deprivation [2, 8, 9]. Diabetes meets all the criteria for disease prevention by virtue of being a chronic disease of major public health consequences with serious complications including microvascular disease such as blindness, kidney failure, and diabetic neuropathy and amputations, together with cardiovascular disease including premature coronary artery disease and stroke causing death to millions of people around the globe [10–13]. Diabetes has also a known predisease stage that is prediabetes (dysglycemia) that necessitates intervention to prevent the disease [12]. It is very important to note that prediabetes carries a high cardiovascular disease (CVD) risk, compared with a nondiabetic population, and therefore makes a compelling case for disease prevention as a public health priority [2, 11, 12].

Various interventions have been shown to be effective in the prevention of type 2 DM, in the prediabetic populations [2, 14]. These include dietary modification, weight loss, increased physical activity as well as drug interventions, and surgical weight reduction among the morbidly obese patients with diabetes [2, 14]. However, lifestyle modification has shown consistently to be more effective (about 60% reduction in diabetes), compared with medication use. For example, in the landmark study, the diabetes prevention program (DDP), lifestyle interventions prevented diabetes by 57%, compared with metformin (32% prevention only) [14–16]. Furthermore, these interventions have shown to be safe, cost effective, and reproducible and have more long-term beneficial effects on CVD risk factors [14–16]. These results have been replicated in other populations with almost identical results including the Finnish diabetes study [17, 18]. Despite these compelling data, long-term adherence to lifestyle modifications poses a challenge, particularly among ethnic minority populations [19].

In this special issue, papers focused on the effects of lifestyle modifications and dietary habits on the prevention of diabetes and cardiovascular disease. Articles covered a wide range of important and topical questions in the field of diabetes prevention as well as the prevention of complications of the disease. One major theme addressed in the special issue was the need to understand and find tailored interventions to reduce diabetes and related complications at the population level. One article that addressed this directly was “Differential and Combined Effects of Physical Activity Profiles and Prohealth Behaviors on Diabetes Prevalence among Blacks and Whites in the US Population: A Novel Bayesian Belief Network Machine Learning Analysis” which presented a novel Bayesian belief network machine learning analysis highlighting the need to provide alternative and personalized behavioral/lifestyle recommendations to generic national physical activity recommendations, addressed by A. Seixas et al. A secondary theme addressed in this special issue was the potentially dual role of prohealth behaviors, such as smoking cessation, as it is linked with CVD and diabetes risk reduction, although it may also be linked to weight gain, a risk factor for diabetes and CVD. K.-W. Kim et al. reported on the association between the time since smoking cessation and insulin resistance, addressed among asymptomatic male Korean ex-smokers, which helped answer some of the questions in this area. A third theme addressed the importance of preventing preclinical risk indicators such as prediabetes, which G. Kerrison et al. addressed in their systematic review on the effectiveness of lifestyle adaptation for the prevention of prediabetes among adults. This is particularly important and goes beyond prevention of diabetes given that the prediabetes state is a CVD risk in itself, as discussed earlier in this paper. A fourth theme directly addressed the effects of dietary modification on the CVD risk and clinical indicators (e.g., stroke), which was evidenced in C. E. van den Brom et al.'s article “Reducing Caloric Intake Prevents Ischemic Injury and Myocardial Dysfunction and Affects Anesthetic Cardioprotection in Type 2 Diabetic Rats.” Finally, the issue of whether vulnerable populations such as racial/ethnic minority groups are more susceptible to diabetes and poorer diabetes management outcomes was addressed in the manuscript “Case Finding and Medical Treatment of Type 2 Diabetes among Different Ethnic Minority Groups: The HELIUS Study,” a particularly vulnerable population for type 2 diabetes and its complications using a multiethnic dataset from the HELIUS Study.

In conclusion, although our special issue does provide novel insights into how biobehavioral and lifestyle factors are associated or contribute to diabetes risk, there are uncharted areas that should be explored in future research. These include the role of health behaviors and lifestyle factors in epigenetics (how environment affects biology and genes), omics (genomics, proteomics, and metabolomics), and precision medicine approaches in the development and maintenance of diabetes and cardiometabolic conditions.

## Figures and Tables

**Table 1:** Risk factors for type 2 diabetes.

Nonmodifiable
(i) Increased age (>45 years) (ii) Genetic factors such as family history of type 2 diabetes (parents or siblings) (iii) Ethnic minorities such as African-Americans, Native Americans, Asian-Americans, and Pacific Islanders (iv) History of gestational diabetes
Potentially modifiable
(i) Sedentary lifestyle, high-caloric, high-fat intake, and high carbohydrate diets (ii) Hypertension (iii) Dysglycemia (impaired fasting glucose and/or impaired postprandial sugar) (iv) Dyslipidemia (high triglycerides, low HDL cholesterol) (v) Central (intra-abdominal) obesity
